# A conceptual framework on the role of backward integration in sustainable access to malaria intervention commodities in Nigeria

**DOI:** 10.1186/s12936-023-04641-z

**Published:** 2023-07-26

**Authors:** Olugbenga A. Mokuolu, Innocent O. Idachaba, Musibau A. Babatunde, Kafayat O. Suleiman, Toluwani A. Mokuolu, Lukman Lawal, Adenike O. Osofisan

**Affiliations:** 1 Department of Paediatrics and Child Health, College of Health Sciences, University of Ilorin, Ilorin, Kwara State Nigeria; 2 Department of Banking and Finance, Ahmadu Bello University, Zaria, Kaduna State Nigeria; 3 School of Business University of Ibadan, Ibadan, Oyo State Nigeria; 4 Centre for Malaria and Other Tropical Diseases Care, University of Ilorin Teaching Hospital, Ilorin, Kwara State Nigeria

**Keywords:** Malaria, Backward integration, Access, Commodities, Sustainability

## Abstract

**Background:**

Over the last two decades, global stakeholders and the Nigerian government have invested approximately $2 billion in malaria control, reducing parasite prevalence to 23% from 42% to 2010. However, there is a risk that the modest gains will be reversed due to unmet resource gaps. Backward integration is presented in this paper as a viable option for sustainable funding of malaria intervention commodities in Nigeria.

**Methods:**

Following a critical appraisal of the resource profile and malaria expenditure, a conceptual framework on backward integration as a means of ensuring long-term supply of malaria intervention commodities was developed. The study analysed secondary annual data from the National Malaria Elimination Programme to estimate commodity needs for the period 2018–2020, as well as total resources committed and the financial gap.

**Results:**

The funds needed to implement national malaria interventions from 2018 to 2020 totaled US$ 1,122,332,318, of which US$ 531,228,984 (47.3%) were funded. The Nigerian government contributed 2.5%, the Global Fund (26.7%), the President’s Malaria Initiative (16.5%), and the UK Department for International Development (6.2%). The funding shortfall was $591,103,335, or 52.7% of the needs. Various funding scenarios were evaluated for their relative merits and limitations, including advocacy for more external funding, bank borrowing, increased domestic resources, and backward integration.

**Conclusions:**

The study concluded that backward integration should be used, based on a government-led public-private partnership that will increase local production of malaria intervention commodities that are accessible and affordable through market-based demand and supply arrangements.

## Background

Malaria has been identified as the most deadly and life-threatening parasite illness on a global scale. In 2021, for example, an estimated 247 million new cases of malaria and 619,000 fatalities were documented globally, with the African area accounting for around 95 and 96% of these, respectively. Nigeria accounted for 39% of the worldwide malaria load and 31% of the global fatalities [1].

**Fig. 1:**
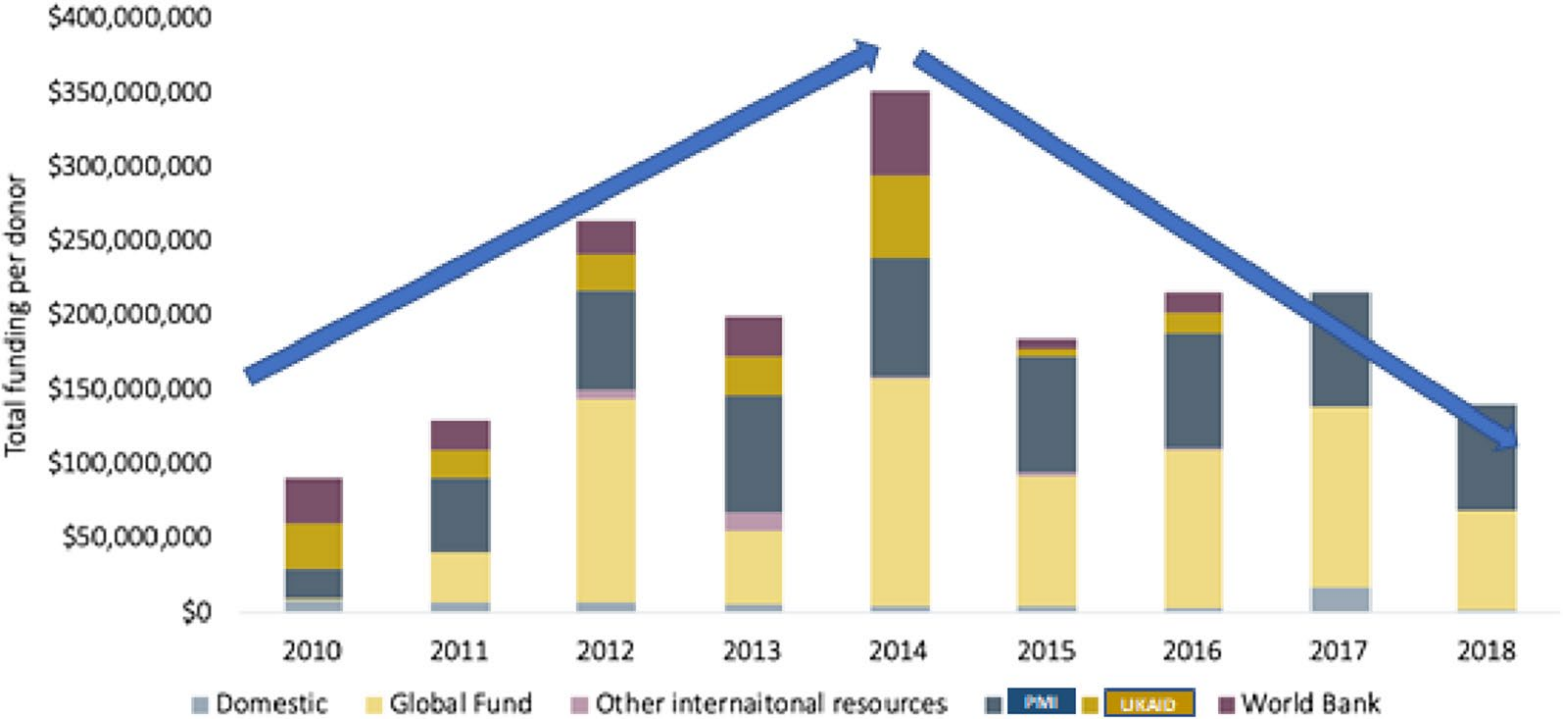
Funding by international agencies

Also, as reported by the World Health Organization (WHO) in 2018, over the last two decades, investment in malaria control from global efforts and the Government of Nigeria is about $2 billion. This has contributed to parasite prevalence dropping from 42% to 2010 to 23% in 2018 and 21% in 2021 [2]. However, as illustrated in Fig. 1, there has been a decline in available funding support to sub-Saharan Africa, particularly Nigeria, since 2015, which has resulted in the threat of reversal of the modest gains due to unmet gaps in the resources needed.

According to Alba et al. [3] the WHO’s malaria control plan is based on timely and effective treatment for all malaria episodes. As a result of this policy, different international aid organizations such as the Global Fund to Fight AIDS, Tuberculosis, and Malaria (GFATM), the World Bank’s Malaria Booster Programme, and Medicines for Malaria Venture (MMV), among others, have accelerated the discovery of new anti-malarials. The public health benefit of such medications; however, is heavily dependent on patients’ capacity to acquire them, and little progress will be made unless larger access concerns are addressed [3]. Despite significant advances in the number of anti-malarial medicines available worldwide, studies performed in sub-Saharan African nations revealed that only 15% of fever cases were treated [4].

Furthermore, numerous techniques for improving access to malaria intervention commodities by focusing on providers or users have been suggested. Among the interventions examined were those aimed at enhancing case management and services at health institutions, as well as those aimed at improving the dispensing practices of drug store attendants and private practitioners, as well as community-based methods and health education campaigns. The review’s major conclusion is that most treatments have been carried out on a limited scale, and only a handful of these programs have been well assessed [5, 6]. As a result, despite a plethora of research in the field, little is known about interventions that can induce long-term transformation.

While scholars have developed several theoretical explanations to justify why malaria intervention commodities have not yielded positive result in sub-Sahara Africa, the widely cited explanation is that the malaria control programme is mainly donor-funded, exposing sub-Saharan African nations, particularly Nigeria, to the risk of donor weariness and significant obstacles to the sustainability of ongoing malaria control and elimination intervention goals [7–10]. A number of previous studies have established that countries especially those in Africa largely depend on the donors given by the international agencies in form of support and concluded that the higher the contribution of the member’s nation, the bigger the support received from the WHO and other agencies [11–15].

Also, there are ongoing doubts in sub-Saharan Africa about the actual number of individuals who die from malaria episodes as a result of medication stock-outs due to a lack of credible statistical data. Nonetheless, the United Nations Children’s Fund (UNICEF) report suggested that insufficient malaria medication supplies are one of the causes of child mortality in sub-Saharan Africa [16]. This indicated a critical need for expedited supply chain coordination at the micro (Nigeria) and macro (sub-Saharan Africa) levels in order to provide access to malaria intervention commodities in endemic nations and ameliorate the status quo.

Furthermore, empirical substantiations of malaria commodity supply and distribution in developing countries, including Nigeria, appear to be difficult, shaped by complex interdependencies among key players such as the Government, donors, patients, interest groups, and private organizations, among others. Nonetheless, the interaction of coordination dimensions and logistics is known to be perennially lacking, as Vledder et al., and Yadav and Rawal assert that few studies on public health have so far investigated supply chain coordination of malaria commodities at the point of service delivery [17, 18].

Multiple lines of evidence have established the role of backward integration in the sustainability of commodities delivery in Nigeria. For instance, over two decades ago, Nigeria’s cement consumption rose to 18.5 million metric tonnes per year ((MMTPA) due to rapid urbanization and industrialization. However, domestic production couldn’t meet the demand, leading to reliance on imports. To address this, the government implemented the backward integration policy in 2002. Under the policy, import licenses for cement were granted only to those building local manufacturing plants, in addition to incentives including waiver of VAT and custom duty for importation of cement production equipment. A study by Elijah assessing the 10 years impact of the policy showed a significant growth, with Nigeria’s cement production increasing from 2 MMTPA to 28 MMTPA. The country now has a total installed capacity of 45 MMTPA and is a major cement exporter to neighboring African countries [19]. Another study by Oshodi revealed that backward integration policy through the use of local raw materials in production significantly led to an increase in manufacturing firms’ value added in Nigeria [20].

However, to the best of researcher’s knowledge, none of the studies have adopted the role of backward integration in sustaining access to malaria intervention commodities. As a result, identifying necessary elements to enable more competent coordination remained critical in the effort to improve responsiveness, flexibility, operational efficiency, and service delivery of malaria commodities in Nigeria. This dilemma significantly aided empirical investigations and subsequent resolutions in the current study on the importance of backward integration as a means of ensuring long-term access to malaria intervention commodities in Nigeria.

As a result, the purpose of this research is to empirically examine the role of backward integration in ensuring long-term access to malaria intervention commodities in Nigeria. Other specific objectives include to:

- Present Nigeria’s estimated quantity and cost of malaria intervention commodities needed for the period 2018 to 2020.
- Present the quantity of the needed commodities that were financed.
- Highlight a funding gap for malaria intervention commodities between the period 2018 to 2020 in Nigeria.
- Propose Business Case towards Sustainable Malaria Intervention Commodities in Nigeria.

### Concept of supply chain management

The vast range of operations necessary to plan, control, and execute a product’s flow from obtaining raw materials and manufacture through distribution to the ultimate consumer in the most efficient and cost-effective manner possible is referred to as supply chain management [21]. For sustainable malaria intervention commodities, supply chain management (integration) should be used, in which the supplier supplies raw materials to the manufacturers, the manufacturers process the raw materials and convert them into finished goods (malaria commodities) by using appropriate modes of transportation to the distributor, the distributor supplies the finished goods to the retailers, and the retailers sell the goods to consumers (patients).

### Pros of backward integration

Backward integration, according to Li and Chen, has several advantages [22]. According to the findings, commercial firms and institutions may engage in integration for the following reasons:

Assurance of timely raw material supply: the firm will be able to maintain an unbroken raw material supply if it is able to manufacture its required raw materials. This eliminates the possibility of a production cycle stoppage and reduces the possibility of variations when comparing production costs to projected costs. The ability to substantially manage the supply of its raw materials through backward integration allows a firm, either directly or through its subsidiaries, to create the quantity of raw materials required for production without or with tolerable delay.

Cost-cutting measures: producing raw materials in-house or through subsidiaries will assist a firm in lowering its manufacturing costs by lowering the cost of resources utilized. This eliminates the supplier’s profit on raw material supply, saving a portion of the company’s projected money outflow.

Standardized and possible good quality raw material: standard is linked with product quality, and it is the attainable quality level that every supervisory body seeks in every product. Standard is the watchword since no firm wants to be caught napping in this area of operation. The quality of raw materials utilised in the production of standard products is the first step. As a result, when a firm manufactures its raw materials, it will be able to identify the degree of quality that will fulfill the requirements of quality manufacturing.

Growth/expansion: the integration programme, whether forward or backward, is an investment in the firm and entails a fund outflow. This represents a direct increase in investment and expansion for the firm.

Variations/experiment: it is customary for firms to change the container of their product in order to physically identify it from others. Only firms having a significant control over the manufacturing of their product’s container (raw material) or who manufacture the container themselves may easily do so without much stress or cost. A firm may also customise a product for a certain occasion or to fulfill specific needs. This is another advantage of backward integration.

Tax reliefs: companies that participate in backward integration programmes with a rural presence are eligible for tax breaks such as the Investment Allowance.

### Theoretical review

The choosing of a theoretical framework on which to base a study is a critical step in the research process. Previous studies on supply chain and malaria intervention commodities over the last decade have led to the development of a number of theories that seek to explain how supply chain can enhance distribution of essential health sectors commodities. The most popularly known theory is the coordination theory. This theory was chosen because of its ability to plausibly justify how supply chain outlets may be responsible for sufficiency in malarial intervention commodities.

### Coordination theory

The Coordination theory as established by Malone and Crowston validated this investigation [23]. The theory proposes that, for greater effectiveness and efficiency, an organization should constantly identify and assign tasks, along with their respective interdependencies (coordination dimensions), meaning that coordinating the supply chain is a critical element that must ensure that all activities in the supply chain are systematically glued together, for superior performance. Malone and Crowston based their hypothesis on the efforts of previous theorists in the domain of supply-chain coordination—as will be discussed more below.

### Coordination theory and malaria commodities

Malaria intervention commodities are key items in any health system, and as such, their supply chain coordination (through backward integration) should be prioritized. Malaria supply networks are complicated and distinct from manufacturing chains in that they often feature vast and prolonged pipelines that necessitate high levels of product availability in the face of significant volatility in supply and demand. As a result, the necessity to analyse and recognize operations (integration) in the health care sector in order to improve decision-support frameworks remains paramount. Regrettably, as climes continue to seek improved health supply chains, relevance is sometimes seen to be quite limited, posing unnecessary problems such as: lack of coordination, absence of demand information, unavailability of companies producing malaria commodities, inadequate engagement of private sector, poor resource profile, ineffective coordination of finance across institutions and initiatives, supply chain challenges, and problems harmonizing the goals of different parties. The coordination theory addresses and contains these difficulties, which is why it is relevant to the current study.

## Methods

The study adopted cross-sectional research design that focused on the projected national needs and resources pipeline for malaria intervention commodities for the period 2018 to 2020. The period was selected to align with the current funding request cycle by donor agencies. Furthermore, the population focused on the entire Nigerians since the objective is attaining universal coverage (100%).

Data for this study was secondary in nature obtained from the National Malaria Elimination Programme (NMEP) showing the quantity of commodities needed for programme implementation, such as artemisinin-based combination therapy (ACT), Rapid diagnostic test (RDT) Kits, Artesunate injection vials, Rectal Artesunate Suppositories (RAS), Long-lasting insecticidal nets (LLINs), Sulfadoxine–pyrimethamine (SP) tablets and sulfadoxine–pyrimethamine + artesunate–amodiaquine (SP–AQ) (FMoH, personal communication). Furthermore, the study was keyed into excel spreadsheet for analysis where percentage, tables and pie-charts were selected and analysed to examine the nexus between malaria intervention and financial gaps.

## Results and discussion

### Malaria commodity needs in Nigeria (2018–2020)

Captured in results are the cost profiles for the commodities needed for malaria intervention in the period under consideration. Table one describes the overall malaria commodity needs of the country over the stated period showing a projected sum of $798,072,138 (USD). The case management items constitute (58%) while the vector control and other preventive commodities constitute (42%), as shown in Table 1. These figures however represent the irreducible minimum with regards to malaria commodity needs. As the country presses towards malaria elimination, and the need to expand the coverage of interventions while also adopting new strategies, the overall need is going to increase significantly, making the business case for malaria to be a multibillion-dollar investment need.

**Table 1:** Malaria commodities need in Nigeria (2018–2020)

Commodity items	2018 (USD)	2019 (USD)	2020 (USD)	Total (USD)
i. Case management items				
ACT-public	34,026,675	35,142,750	35,098,880	104,268,305
ACT-community	3,805,615	3,930,439	3,925,533	11,661,587
ACT-private	74,097,563	76,527,963	76,432,429	227,057,955
RDT-public	29,556,861	30,183,334	35,424,258	95,164,453
RDT-community	369,000	381,103	524,804	1,274,907
RDT-private	3,320,996	3,772,917	11,335,763	18,429,676
Injection-artesunate	1,932,081	1,878,074	1,939,675	5,749,830
Rectal artesunate	189,420	195,633	202,049	587,102
				**Sum (464,193,815)**
ii. Vector control and other preventive commodities				
LLINs-MC	39,501,206	55,870,575	34,245,795	129,617,576
LLINs-RD	10,906,070	11,434,039	11,984,570	34,324,679
Sulfadoxine–pyrimethemine	16,416,756	17,846,129	19,583,245	53,846,130
SP–AQ	21,087,775	47,077,549	47,924,614	116,089,938
				Sum (333,878,323)
Total	235,210,018	284,240,505	278,621,615	798,072,138

Source: (researcher’s compilation from National Malaria Elimination Programme, Federal Ministry of Health, 2008, NMEP gap analysis table for global fund new funding mechanism, personal communication)

*LLINs* Long Lasting Insecticidal Net, *ACT* artemisinin combination therapy, *RDT* rapid diagnostic test, *MC* mass campaign, *RD* routine distribution

## Financial gap analysis of malaria (2018–2020)

**Fig. 2:**
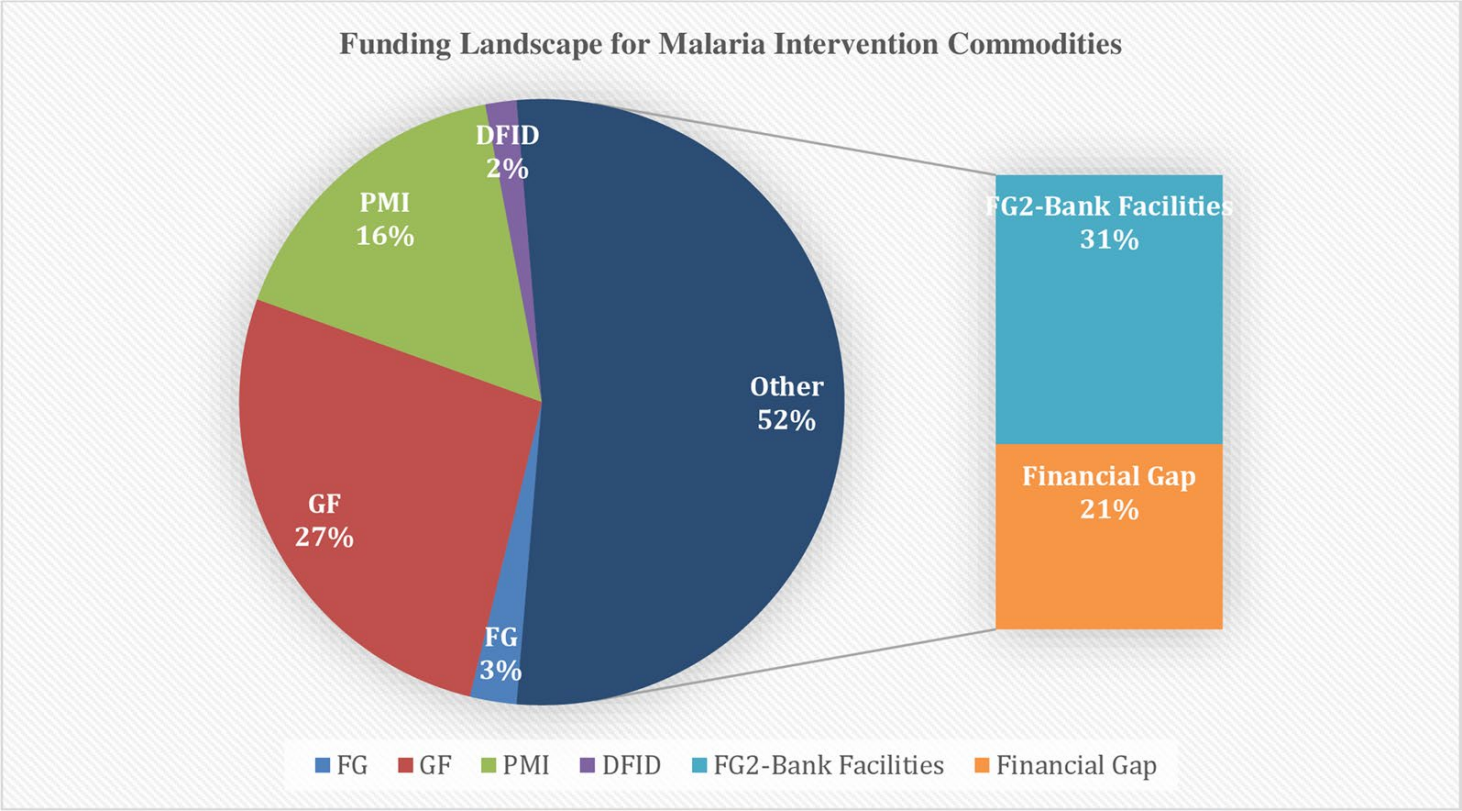
Funding landscape for malaria intervention commodities

Table 2 on the other hand shows the quantity of the needed commodities that were financed. Overall sum of USD 303,541,219 has been provided by the Government of Nigeria (GoN) and other partners, with a financial gap of USD 494,530,919. The contributions are GoN (52%), GF (27%), PMI (16%), DFID (2%), Bank Facilities (31%), as illustrated in Fig. 2. An analysis of the financial gap on an annual basis is shown in Table 3. The gap for each of the years from 2018, 2019 and 2020 were USD 136,609,541, USD 165,368,125, USD 213,484,287, respectively. The challenge of partially implemented activities is the fact that it may render other investment ineffective and create a loss of confidence in the program implementation. The critical question to address is the options for meeting these gaps and the sustainability of the funding for commodities currently financed when the current cycle of planning is over. This challenge remains recurrent with every planning and funding cycle.

**Table 2:** Commodity needs and quantity financed (2018–2020)

Commodity	Total need (USD)	FG (USD)	GF (USD)	PMI (USD)	DFID (USD)	Commodity financed (USD)	Gap in commodity (USD)
LLINs-MC	129,617,576	4,250,000	48,948,343	35,171,442	–	88,369,785	41,247,790
LLIN-RD	34,324,680	360,000	11,740,665	2,717,625	1,246,009	16,064,299	18,260,381
ACT-public	104,268,306	3,000,000	45,268,006	21,187,202	2,695,415	72,150,623	32,117,682
ACT-community	11,661,587		3,558,585		–	3,558,585	8,103,002
ACT-private	227,057,955				–		227,057,955
RDT-public	95,164,453	1,200,000	38,597,850	33,600,000	5,764,255	79,162,105	16,002,348
RDT-community	1,274,906		586,457		–	586,457	688,449
RDT-private	18,429,675				–		18,429,675
Injection-artesunate	5,749,831		1,091,017	800,370	124,562	2,015,948	3,733,882
Rectal artesunate	587,102				–		587,102
SP-IPTp	53,846,131	23,923,066		6,000,000	–	29,923,066	23,923,065
SPAQ-SMC	116,089,939		1,353,316	1,689,000	8,668,035	11,710,351	104,379,588
Total	798,072,141	32,733,066	151,144,239	101,165,639	18,498,276	303,541,219	494,530,919

Source: (researcher’s compilation from National Malaria Elimination Programme, Federal Ministry of Health, 2008, NMEP gap analysis table for global fund new funding mechanism, personal communication)

*IPTp* intermittent preventive treatment for malaria in pregnancy, *SMC* Seasonal Malaria Chemoprevention

**Table 3:** Financial gap analysis of malaria (2018–2020)

Product	2018 (USD)	2019 (USD)	2020 (USD)	Total (USD)
LLINs-MC	12,837,720	14,086,817	18,573,253	45,497,790
LLIN-RD	5,459,780	5,470,368	7,330,232	18,260,380
ACT-public	8,187,202	5,977,085	17,953,395	32,117,682
ACT-community	2,055,032	2,122,437	3,925,533	8,103,002
ACT-private	74,097,563	76,527,963	76,432,429	227,057,955
RDT-public	1,209,035	1,276,307	18,487,690	20,973,032
RDT-community	199,260	205,795	283,394	688,449
RDT-private	3,320,996	3,772,917	11,335,763	18,429,676
Injection artrsunate	1,257,380	1,232,190	1,244,312	3,733,882
Rectal artesunate	189,420	195,633	202,049	587,102
SP-IPTp	6,708,378	7,423,064	9,791,623	23,923,065
SPAQ-SMC	21,087,775	47,077,549	47,924,614	116,089,938
Total	136,609,541	165,368,125	213,484,287	515,461,953

Source: Researcher’s compilation from National Malaria Elimination Programme, 2021

### The percentage contribution of domestic financing of malaria commodities needs in proportion of the total commodities financed

**Fig. 3:**
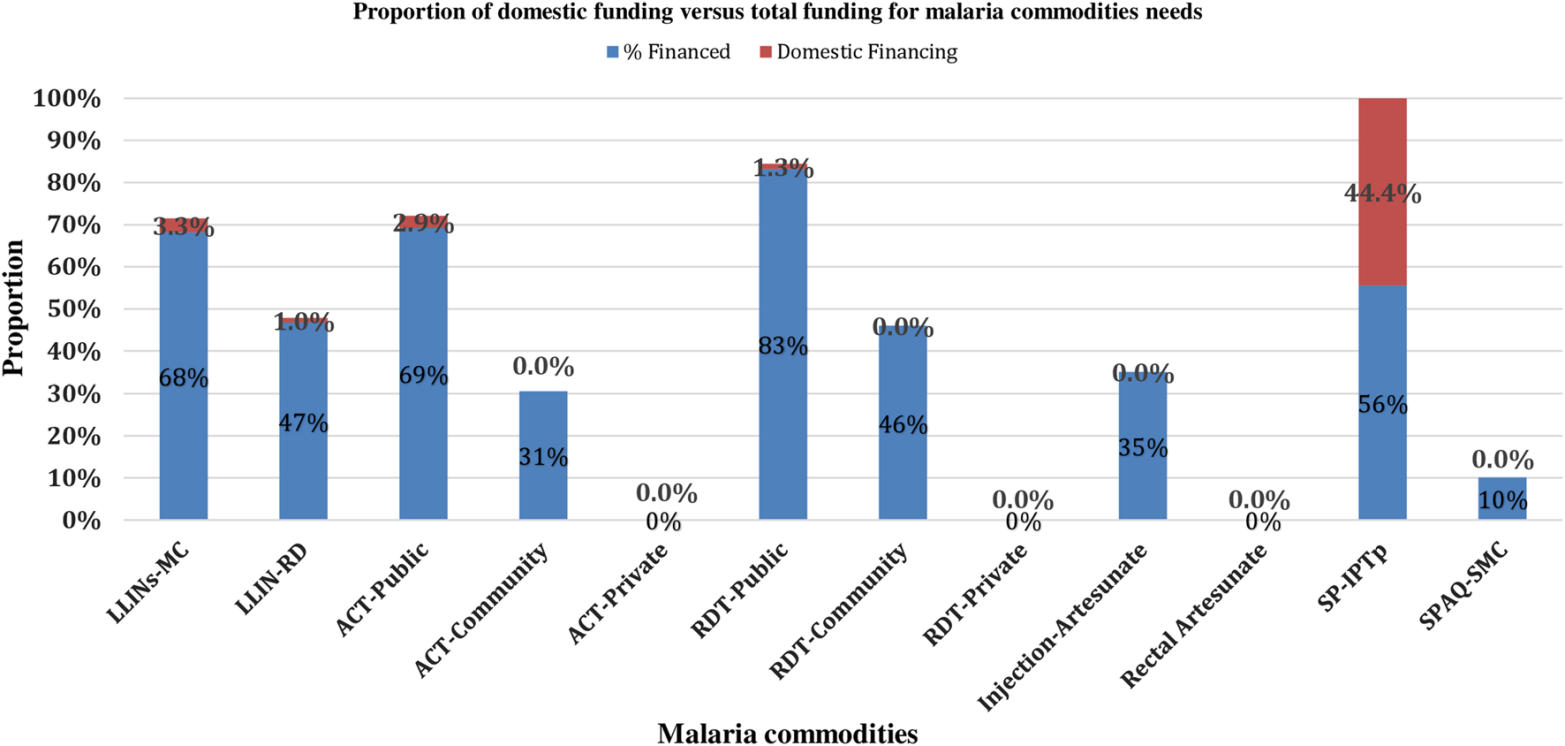
The percentage contribution of domestic financing of malaria commodities needs in proportion of the total commodities financed

Figure 3 shows the percentage contribution of domestic financing of malaria commodities needs in proportion of the total commodities financed. Looking at it critically, the domestic source of funding has not been able to generate more resources over the projected years. For instance, of the total commodities need for ACT-Private, RDT-Private, Rectal Artesunate and SPAQ-Seasonal Malaria Chemoprevention (SMC), none of the resources was sourced domestically. This further created a niche in the gap in the programmatic commodities needs of malaria.

### Proposed business case towards sustainable malaria intervention commodities in Nigeria

The above presentation of needs and resource profile highlight the persistence of financial gaps in meeting malaria intervention commodities’ needs. To address this, there are various funding options that has to be evaluated to guide governments’ efforts at ensuring optimum programmes implementation. A four-case scenario analysis is adopted for this consideration.

#### Scenario 1: Advocacy for more external resources

Donor financing for malaria control has grown significantly, and the proportion of the world’s population living in malaria-free zones has expanded exponentially. Theoretically, with enough international and domestic financing, it is possible to address the malaria burden in an effective, scalable, and sustainable manner. However, partners are increasingly getting fatigue as expressed by the reduction in the funding envelope by some of the institutions. There is now a call for increase domestic resource mobilization as part of the exit strategies for many donor agencies and to ensure sustainability [24, 25]. The implication of the foregoing is that while efforts to increase external resources may grant some respite in the short term, they are unlikely to be sustainable and may therefore fall short of the desired goal of malaria elimination.

#### Scenario 2: use of financial institutions’ instruments

Governments throughout the globe may employ financial institutions to fill a market vacuum and expedite breakthrough solutions that are vital to fighting malaria and protecting global public health. However, borrowing either internally or externally is a major impediment to the growth and stability of the economy. Similarly, borrowing leads to acute external debt burden which are likely to crowd-out investment. Also, borrowing to finance the control of malaria can lead to huge debt burden with its attendant problems. The use of bank facilities can only be justified at best as a temporary intervention to mitigate against potential reversals of previous gains in malaria control. Hence the resource can be used to bridge commodity gap on a short-term basis. Again, this therefore will not be the most logical solution to addressing the financial and commodity gaps in the long run.

#### Scenario 3: increased domestic resources

This is currently viewed as the ultimate solution to addressing the gaps in malaria commodity needs [24, 25]. The High Burden to High Impact (HBHI) initiative of the WHO has four pillars of which the first pillar addresses political will which ultimately is expressed through financial commitments. Increasing domestic resources included improved budgetary allocations, pool funding such as the national health insurance schemes, mobilizing the private sector for philanthropic activities and getting households to take greater responsibilities. Nigeria as a country continues to suffer a high disease burden and is in danger of losing external financing due to sluggish economic development and the associated donor requests for increased government contribution [25]. The ensuing budgetary deficit will have to be filled on a domestic level. The Government of Nigeria and sub-Saharan Africa can establish a basic health care provision fund for example, $50 million annually to basic health care. However, challenges in the public finance management systems of the health sector include general lack of strategic direction in budgeting. While this strategy is laudable and holds a great promise, many of the leading countries contributing the greatest burden of malaria morbidity and mortality are themselves in great financial straits. Meeting a number of their domestic obligations, such as salaries of workers, basic health needs, basic education and maintenance of peace have been challenging [25]. It is therefore important to have other complimentary innovative approaches to expand the concept of domestic resource mobilization.

#### Scenario 4: backward integration approach through public-private partnership

As indicated earlier, backward integration is a form of vertical integration in which a business essentially acquired the supply chain linked to that business thereby promoting efficiency, leveraging on economies of scale and reducing the impact of the negative forces of competition on the supply end [26]. The business case is hereby made for backward integration for a sustainable supply of malaria commodities. This is a more proactive scenario in which the government (as the business owner for the malaria service delivery space) provide the oversight and leadership for an enabling environment to make the private sector thrive in production and distribution of malaria intervention commodities. In this arrangement, government, through the demonstration of strong political will, enunciates appropriate policy environment to protect investors in malaria market. The investors in turn take advantage of government incentives (protected market, tax relief, loan guarantees and coordinated approvals from regulatory authorities) to reduce cost of production of malaria commodities in producing affordable but quality commodities which are sold and distributed through private sector channels to the teaming population in need of the commodities. The government may make some direct procurement for the most vulnerable population to receive the commodities at no charge at all (in form of free interest loan, capacity building, tax relief, and conducive environment among others) and seed funds to enhance local production of anti-malarial commodities.

**Fig. 4:**
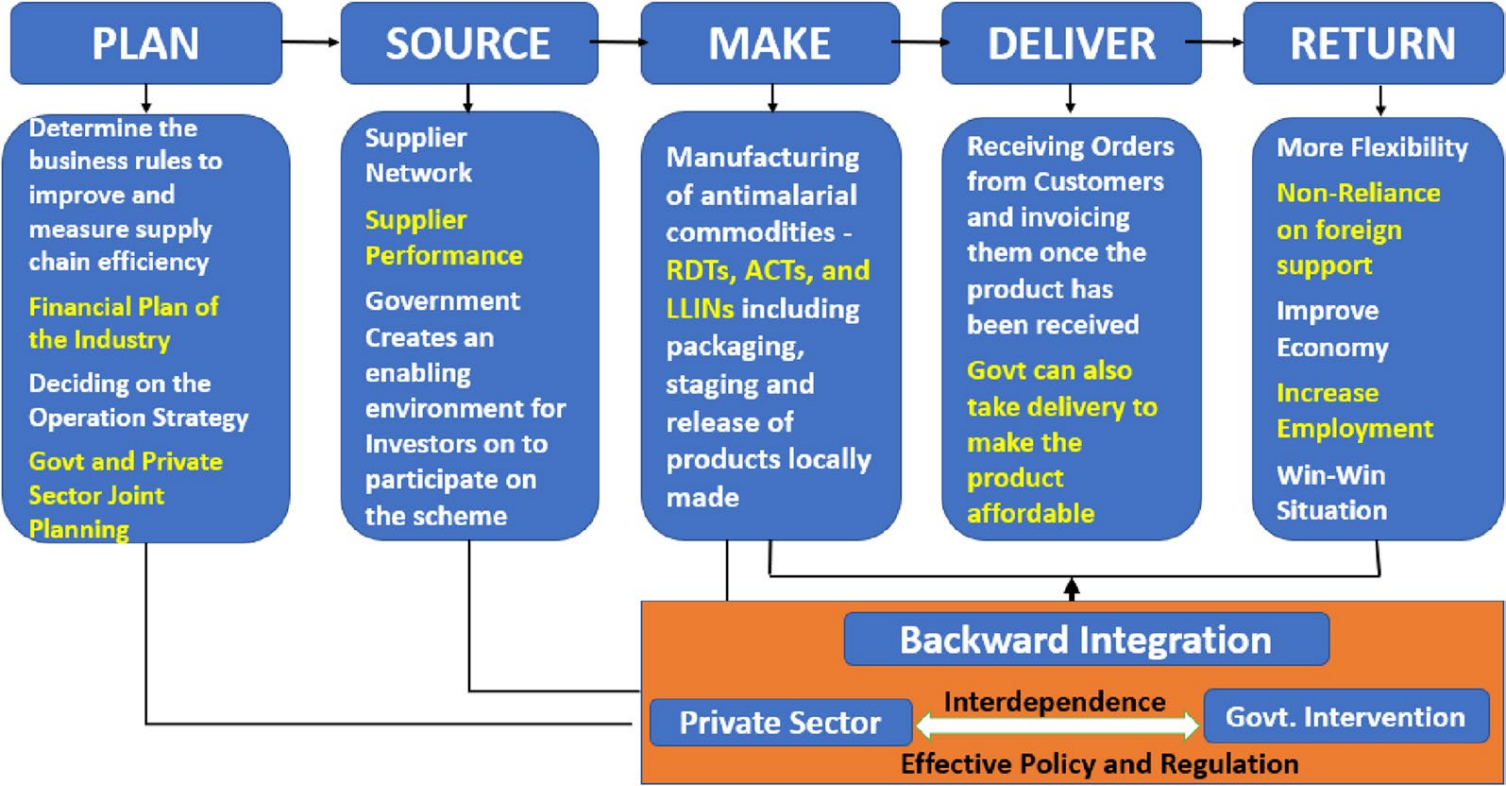
Environmental management processes for backward integration approach

The conceptual framework for this model is provided in Fig. 4. This framework identifies the following phases.

- Phase I (planning): this phase will develop the business rules to improve and measure supply chain efficiency, determine financial plan for the industry and operational strategy.
- Phase II (sourcing): this will include identification of the supplier network, supplier performance with government, creating an enabling environment for investors’ participation. The Government also need to attract high net-worth individuals and relevant private entities to invest in distribution channels to the final consumers.
- Phase III (manufacturing): this will involve the in-country manufacturing of anti-malarial commodities, such as RDT, ACT, LLIN, chemo-preventive therapies among others, with the overall cost and quality meeting the global standard. Innovation and growth can be sustained by ensuring healthy competition between the stakeholders in the market.
- Phase IV (delivery): the exchange between consumers and manufacturers of the commodities. Government may also take delivery for special interventions while boosting the business economy through bulk purchases. The Government can further expand the range of distribution by liaising with the local and global funders of malaria control projects to procure malaria commodities locally.
- Phase V (returns on investment): the country becomes self-reliant and there is minimum importation. The country may export to other countries resulting in a win-win situation.

## Conclusion

Adopting the concept of backward integration will allow for sustainability of the malaria control programme as the supply chain including production, distribution, logistics and design would be under the control of the Government in partnership with the private sector. This will also allow for the consumers to have access to quality products and at very affordable prices. The use of backward integration, based on a government led public-private partnership will enhance local production of malaria intervention commodities that are accessible and affordable using a market-based demand and supply arrangements.

## Data Availability

All data generated or analysed during this study are included in this published article and its Additional files. The data including text, tables, and figures are available for public access without restriction.

## Abbreviations

WHO: World Health Organization
GFATM: Global Fund to Fight AIDS, Tuberculosis, and Malaria
MMV: Medicines for Malaria Venture
UNICEF: United Nations Children’s Fund
NMEP: National Malaria Elimination Programme
ACT: Artemisinin-based combination therapy
RDT: Rapid Diagnostic Test Kits
RAS: Rectal Artesunate Suppositories
LLIN: Long-lasting insecticidal net
SP: Sulfadoxine–pyrimethamine
SP–AQ: Sulfadoxine–pyrimethamine + artesunate–amodiaquine
GoN: Government of Nigeria
SMC: Seasonal Malaria Chemoprevention
HBHI: High Burden to High Impact
